# Fungal diversity on brewery filling hall surfaces and quality control samples

**DOI:** 10.1002/yea.3687

**Published:** 2022-01-12

**Authors:** Elina Sohlberg, Tuija Sarlin, Riikka Juvonen

**Affiliations:** ^1^ VTT Technical Research Centre of Finland Espoo Finland

**Keywords:** beer, brewery, filamentous fungi, filling line, fungal diversity, NGS, nonalcoholic beverage, quality control, spoilage, yeast

## Abstract

Breweries produce an increasing selection of beer and nonbeer beverages. Yeast and filamentous fungi may compromise quality and safety of these products in several ways. Recent studies on fungal communities in breweries are scarce and mostly conducted with culture‐dependent methods. We explored fungal diversity in the production of alcoholic and nonalcoholic beverages in four breweries. Samples were taken for next generation sequencing (NGS) at the key contamination sites in 10 filling lines. Moreover, fungal isolates were identified in 68 quality control samples taken from raw materials, filling line surfaces, air, and products. NGS gave a comprehensive view of fungal diversity on filling line surfaces. The surface‐attached communities mainly contained ascomycetous fungi. Depending on the site, the dominant genera included *Candida*, *Saccharomyces*, *Torulaspora*, *Zygosaccharomyces*, *Alternaria*, *Didymella*, and *Exophiala*. Sanger sequencing revealed 28 and 27 species of yeast and filamentous fungi, respectively, among 91 isolates. The most common species *Saccharomyces cerevisiae* , *Zygosaccharomyces rouxii*, and *Wickerhamomuces anomalus* were detected throughout production. Filling line surface and air samples showed the greatest diversity of yeast and filamentous fungi, respectively. The isolates of the most common yeast genera *Candida*, *Pichia*, *Saccharomyces*, and *Wickerhamomyces* showed low spoilage abilities in carbonated, chemically preserved drinks but could grow in products with reduced hurdles. Preservative resistant yeasts were rare, belonging to the species *Dekkera bruxellensis*, *Pichia manschurica*, and *Zygosaccharomyces bailii*. *Penicillium* spp. were dominant filamentous fungi. The results of this study help to evaluate spoilage risks caused by fungal contaminants detected in breweries.

## Graphical abstract

Yeast and filamentous fungi may compromise quality and safety of beverages. We explored fungal diversity in the production of beer and non‐beer beverages in four breweries. Next generation sequencing revealed that fungal communities on filling line surfaces mostly contained diverse ascomycetous yeasts. Fungal species isolated from quality control samples reflected the range of the beverages produced. Common yeast contaminants showed low spoilage abilities in carbonated, chemically preserved drinks, but could take part in the establishment of biofilm on equipment surfaces.

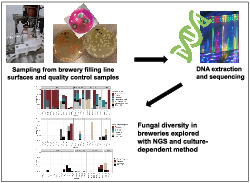

## 1 INTRODUCTION

Despite many advances in controlling microbiological spoilage of beverages, microbial contaminants occasionally cause various defects that are perceptible to a consumer, and liable to cause dissatisfaction, complaint, or rejection of the product. Economic and environmental consequences of the spoilage incidents may be substantial (Begrow, 2017; Stratford, 2006). Fungi, in particular yeasts, are among major spoilage organisms in acid beverages (pH < 4.5) due to their adaptation to acidic habitats containing sugar, alcohol, and/or chemical preservatives and having low oxygen tensions (Juvonen et al., 2011; Stratford, 2006). The category of acid beverages includes a range of alcoholic and nonalcoholic products such as beer, beer mix beverages, ciders, carbonated soft drinks, health and sports drinks, and enhanced waters. Riedl et al. (2019) recently estimated that yeasts cause more than 90% of spoilage incidents in low alcohol beers and beer‐mix beverages. Typical signs of yeast spoilage include swelling and even explosion of packages, development of turbidity, sediments, flocs, or surface films and various off‐flavors described as phenolic, fermented, floral, or vinegar (Hutzler et al., 2012; Stratford & James, 2003). Fermentative yeasts also produce ethanol, which may turn nonalcoholic into an alcoholic drink. The growth of filamentous fungi may lead to formation of hydrolytic enzymes, various off‐flavors and odors, mycelial mats, and discoloration and even allergens and mycotoxins (Filtenborg et al., 2004; Juvonen et al., 2011).

Fungal contaminants may initially find their way into beverage production from various sources such as raw materials, packaging material, insects, and humans (Storgårds & Priha, 2009; Stratford, 2006). Many modern breweries produce a range of beverages, which increases the number of possible microbial sources entering the facility. Microorganisms, including fungi, typically colonize niches that contain moisture and nutrients and are difficult to clean and disinfect (Storgårds & Priha, 2009; Stratford & James, 2003). It has been estimated that 95% of soft drink yeast spoilage incidents are due to poor factory hygiene (van Esch, 1987). In breweries and soft drink factories not using in pack pasteurization, the origin of spoilage incidents can often be traced back to filling machines and the surrounding environment that provide suitable conditions for microbial growth and biofilm formation (Storgårds & Priha, 2009; Timke et al., 2007). Fungi have been found in brewery biofilms and they are among the first microorganisms to attach on filling line surfaces after cleaning (Storgårds et al., 2006; Timke et al., 2005a, 2005b). These pioneer organisms pave the way for other microbes, including spoilage species, but do not necessarily grow in beer themselves.

The past surveys of yeast ecology in breweries and soft drink factories have revealed an extensive list of species (for review, see Hutzler et al., 2012; Stratford, 2006; Stratford & James, 2003). In practice, a limited number of species is able to grow during production or in the final drinks produced under good manufacturing practices. The specific spoilage species are mainly dictated by intrinsic product properties and preservation treatments. In large breweries, beer is mostly brewed with pure yeast, and the term wild yeast is used to refer to any other yeast strain. *Saccharomyces* species, in particular *Saccharomyces cerevisiae* and other *Saccharomyces* sensu stricto species, pose the greatest threat to beer quality both during brewing and in the pack by being strongly fermentative under low oxygen tensions (Hutzler et al., 2012; Kühle & Jespersen, 1998; Pham et al., 2011; Timke et al., 2007). Many aerobic or weakly fermenting *Candida*, *Pichia*, and *Wickerhamomyces* (including some former *Pichia*) species have been identified in brewery raw materials and equipment surfaces (Kühle & Jespersen, 1998; Pham et al., 2011; Storgårds et al., 2006; Timke et al., 2007). They may proliferate during initial phases of fermentation or in final beer in cases of oxygen ingress. Frequently identified species include *Wickerhamomyces anomalus* (former *Pichia anomala*), *Meyerozyma guilliermondii* (former *Pichia guilliermondii*), *Candida sake*, *Candida parapsilosis*, and *Dekkera bruxellensis*. Increasingly popular beer mix beverages, low‐alcohol and nonalcoholic beers, and hop‐reduced beers may support the growth of a wider range of yeasts than “standard” beers (Hutzler et al., 2008).

Yeast contaminants associated with soft drink production have been traditionally classified according to spoilage potential (Davenport, 1996; Hutzler et al., 2012; Stratford & James, 2003). The most dangerous spoilage yeasts in carbonated products include approximately 10–12 fermentative preservative‐tolerant species, the most important being *Zygosaccharomyces* spp. (especially *Zygosaccharomyces bailii*), *Dekkera anomala*, *D. bruxellensis*, *Dekkera naardenenesis*, *S. cerevisiae*, *Kazahstania exigua* (former *Saccharomyces exiguus*), and *Schizosaccahromyces pombe*. In practice, opportunistic species that grow in the product only if some of the intrinsic hurdles are lowered due to errors in manufacturing or high microbial load cause most of the spoilage incidents (Stratford, 2006).

Filamentous fungi contaminations are usually due to poor factory hygiene or due to growth of heat‐resistant species in heat‐processed beverages (Wareing, 2016). They usually originate from outdoor air or soil (Tribst et al., 2009; Wareing, 2016). Any airborne filamentous fungi can contaminate finished products, but vigorously sporulating species are the most common in the soft drink and juice industry (Wareing, 2016). These species belong to genera *Penicillium*, *Aspergillus*, *Eurotium*, *Fusarium*, *Cladosporium*, and *Alternaria*. Heat‐resistant filamentous fungi able to cause spoilage of soft drinks include species from genera *Aspergillus*, *Byssochlamys*, *Peacilomyces*, *Phialaphora*, and *Talaromyces* (Wareing, 2016). They can even survive flash pasteurization of 90°C to 96°C for 30–90 s or tunnel pasteurization of 72°C to 80°C for 5–20 min and can thus grow in pasteurized products (Juvonen et al., 2011). Several spoilage filamentous fungi can grow in low pH, and although they usually require oxygen to grow, some species can grow under anaerobic conditions with fermentative metabolism (Filtenborg et al., 2004).

In response to consumer demands, the diversity of beer and nonbeer beverage products is continuously increasing and traditional hurdles for microorganisms (such as chemical preservatives and alcohol) are being reduced, which is creating new opportunities for microbial contaminations and spoilage and could also affect fungal diversity in this environment (Hutzler et al., 2008; Juvonen et al., 2011). Much of the current knowledge on fungal populations in breweries is relatively old and mainly gathered using cultivation and isolation techniques (Kühle & Jespersen, 1998; Pham et al., 2011; Storgårds et al., 2006; Timke et al., 2007). The present study was undertaken to examine fungal diversity at the key contamination sites of filling lines in four modern breweries with next generation sequencing (NGS). This technique could provide new insight into fungal diversity, providing an opportunity to get a good coverage of the communities with reasonable work (Priha et al., 2016). Moreover, a culture‐dependent method was used to identify various types of fungi in quality control (QC) samples and spoilage potential of selected isolates was evaluated.

## 2 MATERIALS AND METHODS

### 2.1 Fungal community profiling of surface samples

#### 2.1.1 Surface samples

Surface samples were obtained from filling lines in four breweries to assess fungal community composition on the surfaces. The breweries performed the sampling between February and April 2017. In total, 55 samples were collected during the production of various beer and nonbeer beverages. Each brewery provided 6–12 samples from three to four product categories (Table 1). At the time of sampling, the filling lines had been typically operating 1–5 days since last extensive washing. Some breweries performed mild washes during production. Surface area of 10 cm × 10 cm was swapped with sterile nonwoven gauzes (Mesoft, Mölnlycke Health Cara, Gothenburg, Sweden), which were placed immediately in 30 ml of sterile 0.9% sodium chloride (Merck, Darmstadt, Germany) solution. The samples were transported to the molecular biology laboratory in a cold box (+6°C) the same day or at the latest the next morning. Yeasts and filamentous fungi were detached from the gauzes by homogenization for 1 min in a Stomacher blender (Seward Laboratory System, Worthing, UK). Before DNA extraction, the viable counts of filamentous fungi and yeasts in the homogenates were determined on YM agar medium with chloramphenicol (100 mg l^−1^) (PD/Difco, USA).

**TABLE 1:** Surface swipe samples from filling line surfaces for NGS analysis and independent fungal isolates taken from quality control samples for Sanger sequencing. Different breweries marked with A, B, C, and D

Sample type	Sample source	Total number of samples	Number of qPCR‐positive samples by brewery				
			A	B	C	D	Total
Filling line surface swipe samples for NGS	Soft drink	11	4	0	2	0	6
	Beer	20	6	2	0	4	12
	Mineral water	13	1	0	2	0	3
	Other alcoholic beverage	11	0	0	4	0	4

			Number of yeast/mold isolates by brewery				
			A	B	C	D	Total
Quality control cultivation samples	Raw materials	7	5/0	3/0	0/0	2/0	10/0
	Filling hall air	12	0/6	0/9	2/17	0/0	2/32
	Filling line surfaces	12	4/1	0/0	0/0	16/2	20/3
	Soft drink	16	5/1	2/0	13/9	0/0	15/10
	Beer	17	3/0	1/7	0/0	4/5	8/13
	Other alcoholic beverage	4	0/0	1/0	2/1	1/0	4/1

#### 2.1.2 DNA extraction

The homogenized swab sample suspensions were filtered through Sterivex‐GP 0.22‐μm‐pore‐size filter units (Millipore, Billerica, MA, USA) and stored frozen (−80°C) until DNA extraction. Subsequently filters were aseptically cut into smaller pieces and transferred into DNA extraction tubes. DNA was extracted using a Fast DNA Spin Kit for Soil (MP, Biomedicals, USA) according to manufacturer's instructions, with the modification that the cells were homogenized in a FastPrep‐24 instrument (MP, Biomedicals, USA) at 6 m s^−1^ for 3 min. DNA was eluted using 100 μl of DNase/Pyrogen‐free water. Total DNA was quantified using Nanodrop 2000/2000c (Thermo Scientific, USA).

#### 2.1.3 Quantitative fungal PCR analysis

The presence and the amount of fungal DNA was determined with a TaqMan based quantitative PCR (qPCR) method. For the qPCR analysis, Roche LightCycler® 480 Probes Master 2× (Roche Diagnostics, Penzberg, Germany) was applied. The qPCR reactions were performed in 25‐μl reaction volumes containing 2.5 pmol of each primer, 1× Roche LightCycler® 480 Probes Master mix, and 1‐μl template DNA (2.2–24.3 ng μl^−1^). The fungal 5.8S gene region was amplified with primers 5.8F1 5.8R1 and 5.8P1 (Haugland & Vesper, 2002). For detection, 5.8P1 was labeled with the fluorescent dye FAM® (reporter) and TAMRA® (quencher). The qPCR reactions were conducted in triplicate using following protocol in the LightCycler 480 instrument (Roche Diagnostics, Penzberg, Germany): an initial denaturing step at 95°C for 5 min, 40 cycles of amplification step including 10 s at 95°C, 30 s at 60°C and 1 s at 72°C. Cooling for 15 s was performed at 40°C.

#### 2.1.4 Sequencing and sequence analysis

DNA samples containing detectable amounts of fungal DNA were sent for Illumina Miseq sequencing of the fungal ITS2 region to Microsynth AG (Switzerland). As a positive control, a mock DNA sample containing known amount of DNA from six species of yeast *Zygosaccharomycetes bailii* VTT C‐05679, *Zygosaccharomyces rouxii* VTT C‐07807, *D. bruxellensis* VTT C‐05796, *D. anomala* VTT C‐91183, *S. cerevisiae* VTT C‐08823, *Wicherhamomyces anomalus* VTT C‐02462 and one species of filamentous fungus *Trichoderma harzianum* VTT D‐161648 was used. First, ITS amplicon libraries were performed with ITS Nextera two‐step PCR with ITS3/ITS4 primers (ITS3: GCATCGATGAAGAACGCAGC, ITS4: TCCTCCGCTTATTGATATGC) (White et al., 1990). ITS Nextera two‐step PCR with ITS3/ITS4 primers included purification and pooling and Miseq run micro 2*250 v3.

The sequence reads obtained from Illumina Miseq sequencing were subjected to sequence analysis using the DADA2 software package (Callahan et al., 2016) and DADA2 Pipeline Tutorial 1.16 with some modifications. DADA2 package was run in RStudio (version 1.4.1106) with R version 4.0.4. First, the sequences were prefiltered to remove ambiguous bases (Ns) that could affect accurate mapping. The primers were identified from the sequences and removed using a cutadapt tool (Martin, 2011). Quality of the sequence reads was checked according to DADA2 workflow. Next, the sequences were filtered and trimmed using DADA2 filterAndTrim function. Filtering parameters maxN = 0, maxEE = c(2, 2), truncQ = 2, and minLen = 50 were used. Minimum length 50 bp was used to remove spurious very low‐length sequences. The maximum possible error rates were calculated using the learnErrors command. Identical reads were dereplicated (unique sequences). Amplicon sequence variants of the sequence data were identified using DADA2 pipelines core sample inference algorithm. Denoised paired reads were merged according to the DADA2 pipeline and an amplicon sequence variant table (ASV) was constructed. Subsequently, chimeric sequence reads were removed from the data set with remove BimeraDeNovo function, using the consensus option. Finally, taxonomy from domain to genus‐level was assigned to ASVs with DADA2's native implementation of the naive Bayesian classifier method. Taxonomy was assigned against UNITE database version 8.2 (2020‐02‐04) (Kõljalg et al., 2013). All images of the sequencing data were constructed with R using packages the phyloseq (McMurdie & Holmes, 2013) and ggplot2 (Wickham, 2015). Alpha diversity indexes chao1 (Chao, 1984) and Shannon diversity index (Shannon, 1948) were calculated using phyloseq package. Resulted fungal ITS region sequences have been submitted to the European Nucleotide Archive (ENA, https://www.ebi.ac.uk/ena/) under accession numbers ERS7274082‐ERS7274106.

Statistical analysis of NGS data was performed using principal coordinate analysis (PCoA) with R (RStudio Team, 2021) with phyloseq (McMurdie & Holmes, 2013) and ggplot2 (Wickham, 2015).

### 2.2 Isolation and identification of fungi

#### 2.2.1 QC samples

Regular QC samples were obtained from four breweries producing a range of alcoholic and nonalcoholic beverages. The samples were cultivated at the breweries with their own culturing methods and media. The samples included various raw materials (sugar, syrups etc.), surface swabs from filling line equipment, air samples from filling halls, and beverage products (Table 1). Information about the sampling (sampling time, description of the sample, sampling method, sampling point, extra information, e.g., washing of production lines) was collected for each sample. In total, 74 fungi containing liquid enrichment or agar plate samples were obtained. Yeast and filamentous fungi pure cultures were isolated on YM agar medium (PD/Difco, USA). Altogether, 214 pure cultures were recovered consisting of 135 yeast and 79 filamentous fungi isolates.

#### 2.2.2 PCR and Sanger sequencing

DNA from pure cultures was extracted with Fast DNA Spin Kit for Soil (MP Biomedicals, USA) according to manufacturer's instructions with same method as with NGS samples except that yeast colony or fungal mycelium from pure cultures growing on YM agar medium were used as a starting material. For identification of yeast and filamentous fungi pure cultures, PCR products of D1/D2 region for yeasts and fungal ITS region for filamentous fungi were amplified from extracted DNA and sent to SeqLab‐Microsynth (Balgach, Switzerland) for Sanger‐sequencing from both directions of the amplification product. End‐point PCR was performed using primers NLF1F and NLF4R (Kurtzman & Robnett, 1997) for yeasts and ITS1F and ITS4 (Gardes & Bruns, 1993; White et al., 1990) for filamentous fungi. PCR reactions were performed in 50‐μl final reaction volumes, containing 10× Optimized DyNAzyme buffer (Thermo Fisher Scientific, Inc.), 10‐mM final concentration of dNTPs, 25 pmol of each primer, 1.25 units of DyNAzyme II DNA Polymerase (Thermo Fisher Scientific, Inc.), and 5 μl of DNA template. PCR reaction was performed in Eppendorf PCR cycler using following protocol: initial denaturing step at 95°C for 2 min, 35 cycles of amplification step including 1 min at 95°C, 1 min at 52°C and 2 min at 72°C, final extension of 10 min at 72°C and cooling at 12°C.

Sanger sequences were trimmed and forward and reverse sequences were assembled as consensus sequence with Geneious software version 10.2.3 (https://www.geneious.com). Closest yeast and fungal taxonomic matches (98–100%) were achieved from NCBI database with Blast tool (Morgulis et al., 2008) in Geneious software.

### 2.3 Growth tests in commercial beverages

Spoilage ability of selected yeast isolates was studied in commercial beverage products. The products included a simple sugar‐containing soft drink preserved with sorbate (pH 2.9), a preservative‐free juice‐containing soft drink (pH 2.7), a still water product containing sugar and benzoate as major constituents (pH 4.3), a beer with 4.5% (v/v) alcohol (pH 4.2), an apple cider with 4.5% (v/v) alcohol and sulfite (pH 3.0) and a preservative‐free nonbeer beverage with 5.5% (v/v) alcohol (pH 2.8). The products were aseptically distributed in 9‐ml aliquots into 10‐ml plastic screw capped tubes (Greiner bio‐one, Austria). Yeast Mould broth (YM, BD/Difco, USA) was used as growth control medium. The yeast strains were refreshed in YM broth for 1–2 days at 25°C and diluted in Ringer's solution (Merck, Darmstadt, Germany) as required. Each product was inoculated at 10^3^–10^4^ cfu ml^−1^ in duplicate. Inoculated and uninoculated tubes of each product were incubated at 25°C for 6 weeks. Turbidity was assessed every week visually and by measuring turbidity at 620 nm wavelength (Multiskan EX instrument, Thermo Labsystems, Finland). The growth result was scored as no, weak, moderate and intense when the turbidity increase was <1.5‐fold, 1.5 to twofold, more than twofold but less than fourfold, and more than fourfold compared with the uninoculated control, respectively. Samples that were turbid by nature were cultivated on YM agar plates (BD/Difco). At the end of the follow up period, viability and purity of the inoculated yeast strains was confirmed by plate cultivation and final pH was measured. Moreover, the inoculated and noninoculated samples were sniffed by a single person to detect any obvious off‐flavors.

## 3 RESULTS

### 3.1 NGS analysis of fungal communities on filling line surfaces

In total, 55 surface samples from key contamination sites of various filling lines were obtained from four breweries for NGS analysis of fungal communities (Table 1). Fungal DNA was detected with qPCR in 25 out of the 55 samples (Table 1). The highest number of qPCR positive samples derived from beer filling lines (Table 1). Fungal diversity in the qPCR positive samples was studied with Illumina Miseq NGS analysis. Altogether 826,418 filtered fungal sequences were obtained. In total, 203 ASVs were detected in the sample set. The number of observed ASVs in the samples varied between 4 and 48 (Figure S2). When comparing the Chao1 ASV richness estimate values to true observed ASV numbers, all of the estimated fungal ASVs were obtained from the sequence data (Figure S2), meaning that sequencing depth was sufficient to fully characterize the fungal communities in all of the samples.

Fungi from phylum *Ascomycota* dominated fungal communities in all samples (Figure S1). Fungi affiliated to *Basidiomycota* were found in approx. one third of the samples. Their relative abundance exceeded 20% only in four samples. Furthermore, low relative abundancies (<10%) of *Mucoromycota* or unaffiliated fungi were detected in 40% of the samples.

**FIGURE 1:**
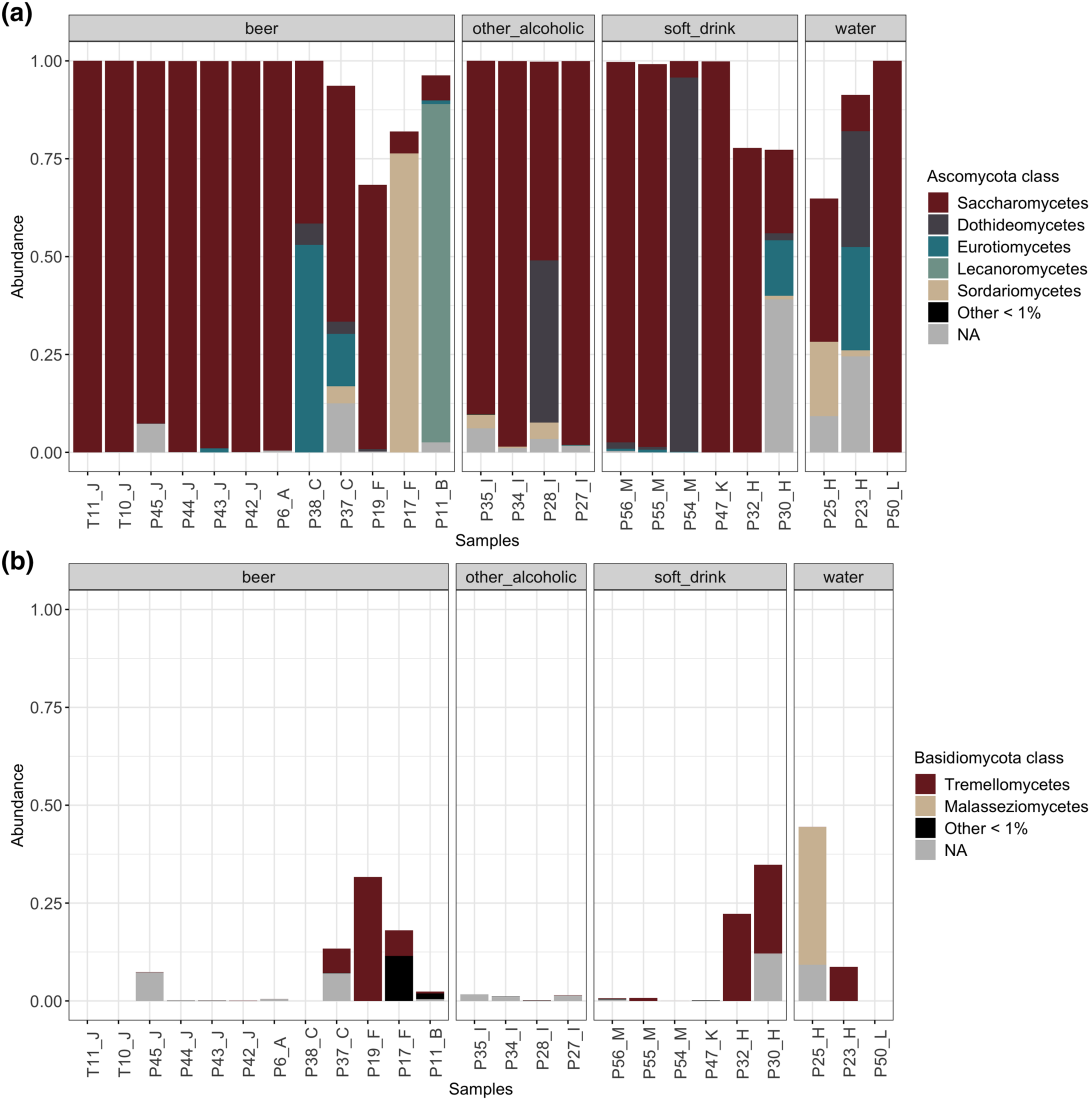
Fungal classes present in brewery bottling and canning line surfaces as determined by NGS. Relative abundances (%) of fungal classes within (a) *Ascomycota* and (b) *Basidiomycota* phylum. Classes detected under 1% abundance are grouped together. NA; fungi not affiliated to any known taxon [Colour figure can be viewed at wileyonlinelibrary.com]

*Saccharomycetes* was the dominating fungal class (relative abundance >50%) in 72% of the samples (Figure 1). Filamentous fungi from the classes *Dothideomycetes*, *Eurotiomycetes*, *Lecaronomycetes*, *Sordariomycete*s, and *Leotiomycetes* were detected in varying relative abundancies, depending on the sample. Basidiomycota on the filling line surfaces mainly belonged to the classes *Malasseziomycetes* and *Tremellomycetes*.

**FIGURE 2:**
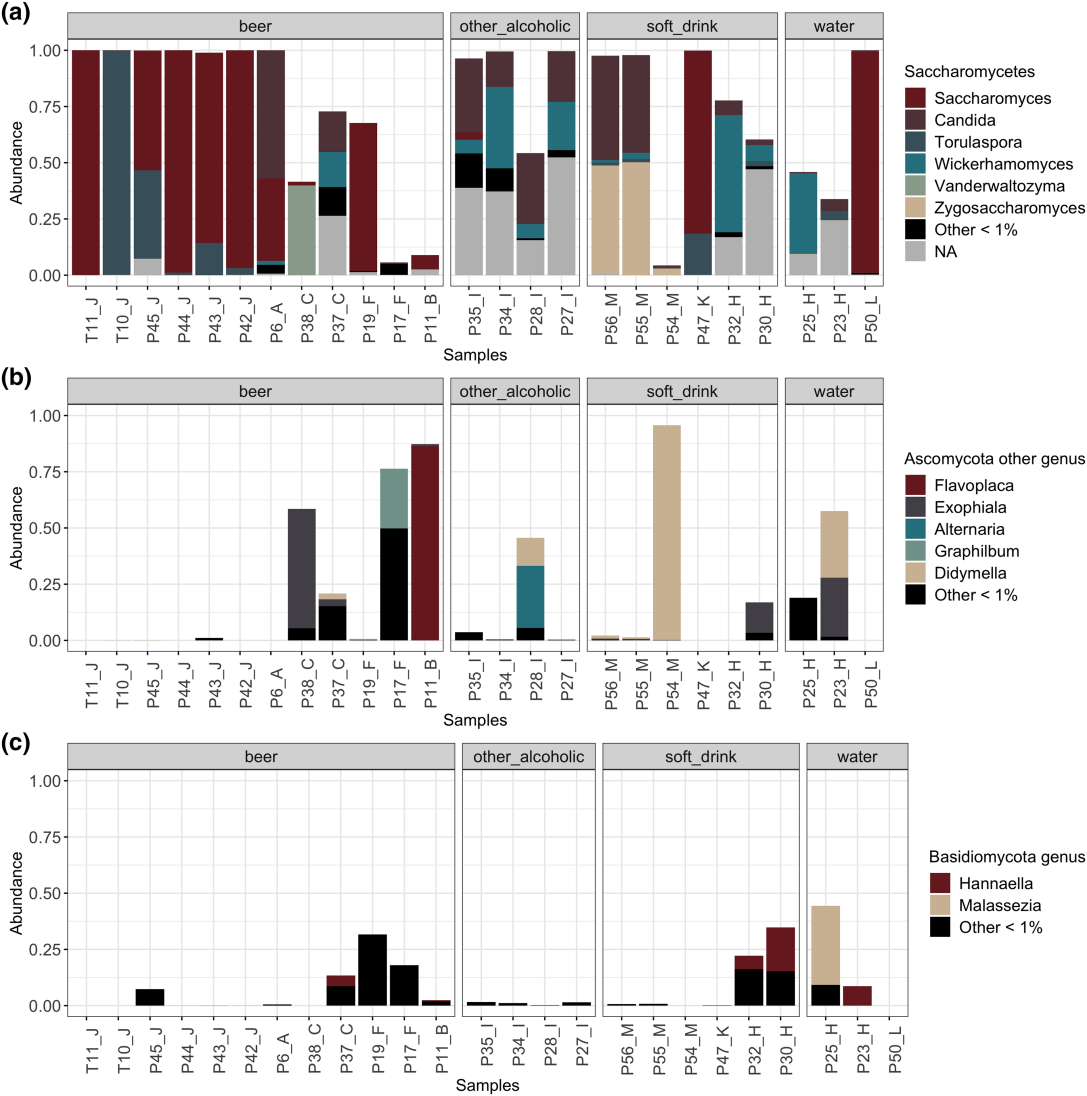
Fungal genera present in brewery bottling and canning line surfaces as determined by NGS. Relative abundances (%) of fungal genera within (a) *Saccharomycetes* class, (b) other *Ascomycota* classes, and (c) *Basidiomycota* phylum. Genera detected under 1% abundance are grouped together NA; fungi not affiliated to any known taxon [Colour figure can be viewed at wileyonlinelibrary.com]

In total, 13 and six ascomycetous yeast genera were detected in the surface samples at above 0.1% and 1% relative abundance, respectively. On average, the principal yeast genera detected were *Saccharomyces* (29%), *Candida* (11.4%), *Wickerhamomyces* (7.5%), *Torulaspora* (7.4%), and *Zygosaccharomyces* (4%) (Figure 2a). Moreover, *Vanderwaltozyma* yeasts were abundant (40%) in one of the samples (P6_A). *Zygosaccharomyces* were only detected in the surface samples taken during soft drink filling.

Other fungi with *Ascomycota* affiliation were distributed between several classes and their relative abundancies varied greatly between the different samples (Figure 2b). Altogether 13 and five genera were identified with relative abundance above 0.1% and 1%, respectively. Genera *Didymella* and *Alternaria* from *Dothideomycetes* class and species from the genus *Exophiala* from the *Eurotiomycetes* class showed the highest abundances. In one sample (P11_B), genus *Flavoplaca* from the class *Lecaronomycetes* had the highest relative abundance.

Among the basidiomycetous fungi, two yeast genera *Hannaella* and *Malassezia* were detected with relative abundance at above 1% (Figure 2b). *Piskurozyma*, *Tremella*, and *Kwoniella* were detected at less than 0.1% relative abundance (data not shown). Basidiomycetous yeasts often co‐occurred with filamentous fungi from the class *Ascomycota* (Figure 2b,c).

**FIGURE 3:**
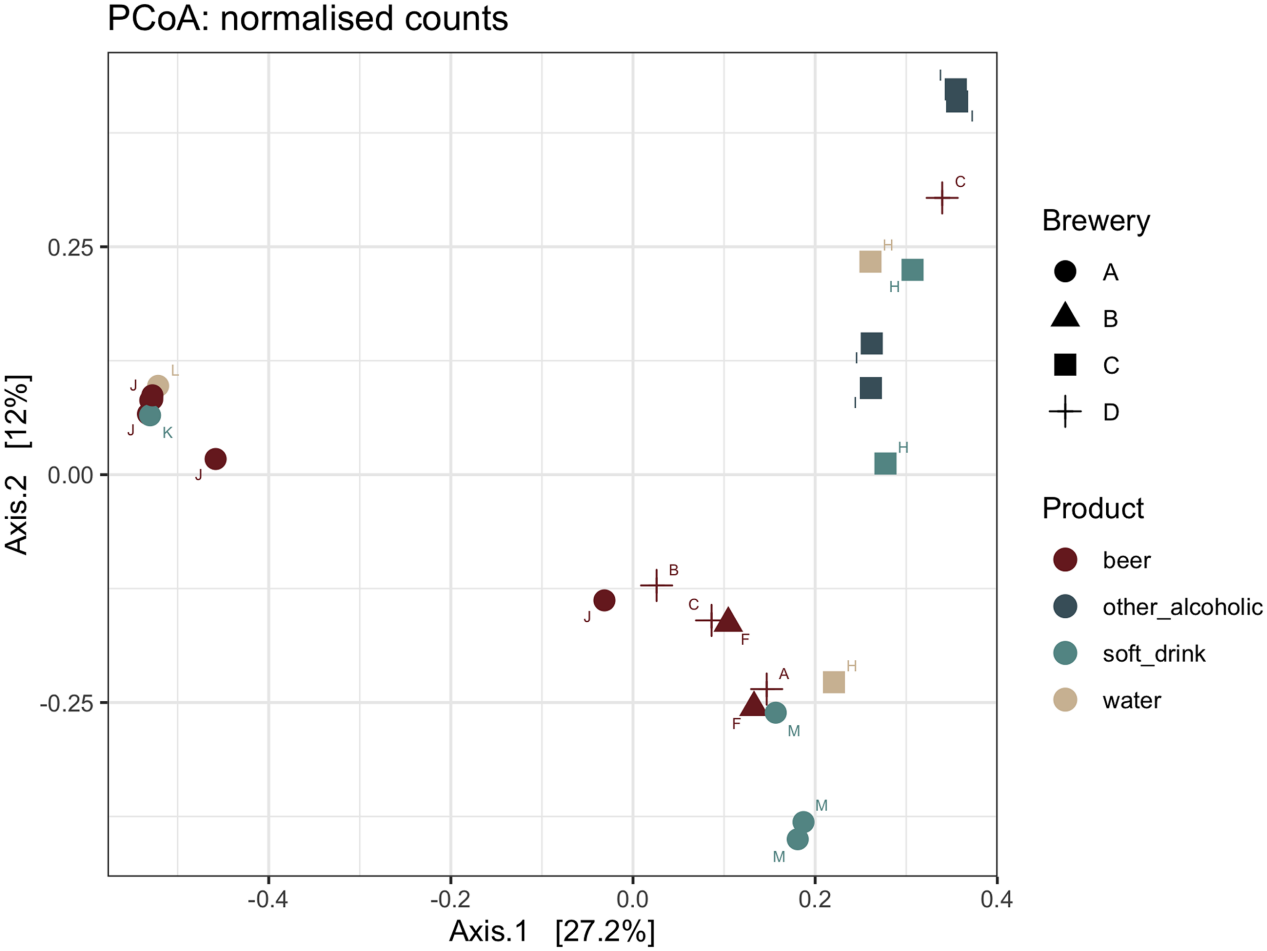
Principal coordinates analysis (PCoA) of brewery bottling and canning line surface fungal community composition based on Bray‐Curtis dissimilarities. Axis 1 explains 27.2% of the variance, whereas Axis 2 explains 12% of the variance. Letters from A to M denote different filling lines [Colour figure can be viewed at wileyonlinelibrary.com]

Microbial community resemblance in the surface samples was studied by performing PCoA analysis based on the relative abundance of ASVs in the samples (Figure 3). Hierarchical clustering of the surface samples on the basis of the first two principal components, coarsely separated three clusters: samples from brewery A filling lines with high abundance of *Saccharomyces* yeasts, samples from brewery C filling lines dominated with *Candida*, *Wickerhamomyces*, and unaffiliated yeasts, and samples from various breweries with mixed community of yeasts and other fungi (Figure 2). n the majority of brewery A samples, cultivable fungi were not detected (data not shown).

### 3.2 Isolation and identification of yeasts and filamentous fungi in QC samples

Morphologically different filamentous fungi and yeast colonies were isolated from 68 QC samples and subjected to Sanger sequencing of ITS and D1/D2 regions. The fungi containing culture samples included seven raw material, 12 air and 12 filling line surface samples, and 37 product samples.

**FIGURE 4:**
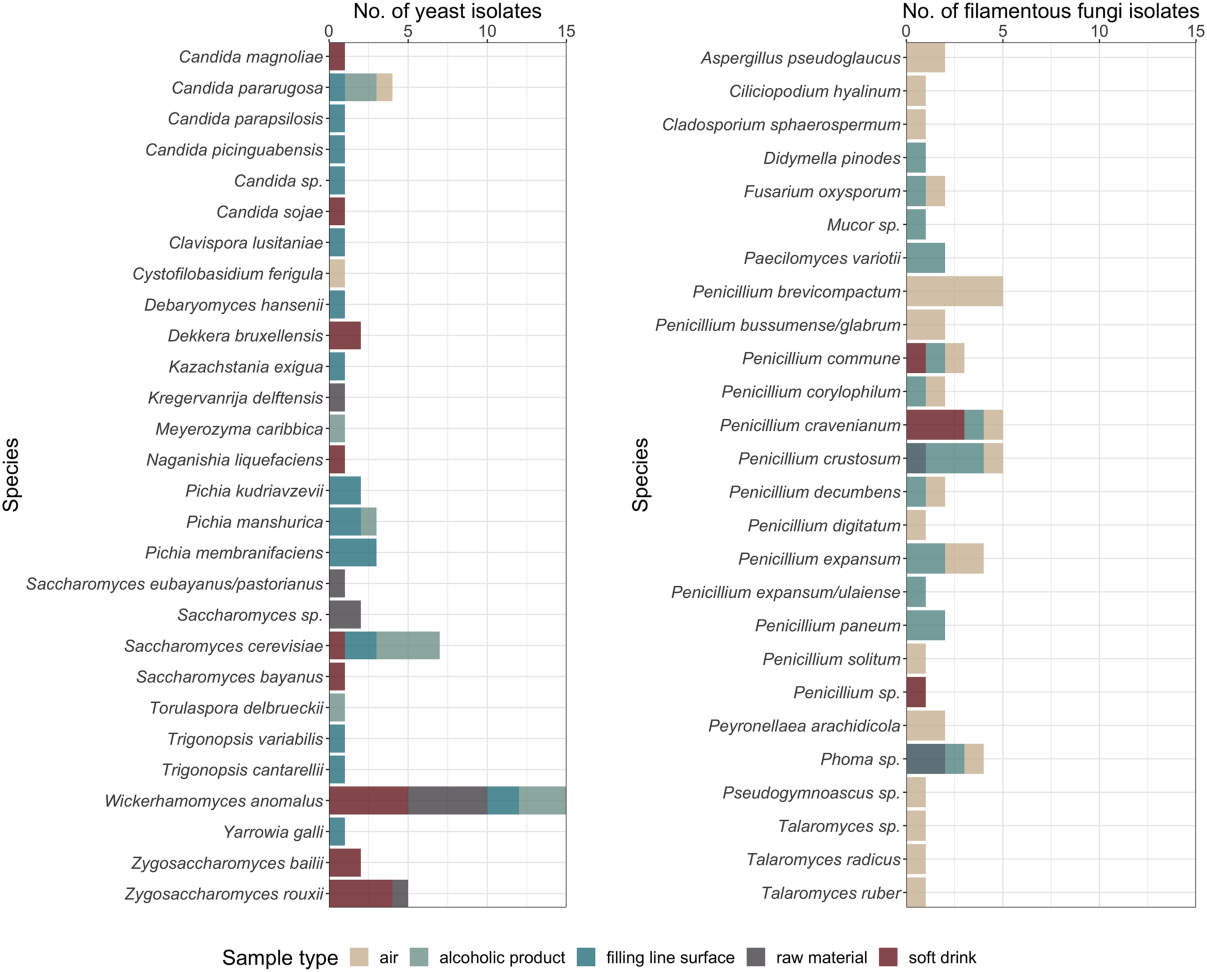
Identification and number of yeast and filamentous fungi isolates isolated from QC samples taken from air, alcoholic products, filling line surfaces, raw materials and soft drinks of four breweries. Identification was carried out with Sanger sequencing of the D1/D2 region for yeasts and ITS region for filamentous fungi [Colour figure can be viewed at wileyonlinelibrary.com]

The 59 independent yeast isolates identified from QC samples were distributed between 14 *Ascomycota* and two *Basidiomycota* genera (Figure 4a, Table S1). Overall, the most prevalent genera in the samples (% of the isolates) were *Wickerhamomyces* (24.6%), *Candida* (14.8%), *Saccharomyces* (14.8%), *Pichia* (13.1%), and *Zygosaccharomyces* (11%). Sixty‐one strains could be identified below genus level and they represented 26 species or species groups. Of the various species/species groups, 64% were represented by a single isolate. The highest number of independent isolates were identified as belonging to the species *W. anomalus* (23.4%), followed by *S. cerevisiae* (10.9%), *Z. rouxii* (7.8%), and *Candida pararugosa* (6.3%).

*W. anomalus* and *Saccharomyces* yeasts were isolated across the various sample categories. Some differences in the species distribution and richness between the various sample categories were found. The greatest diversity, altogether 14 different species, was detected in the filling line surface samples. Various *Pichia* and *Candida* species were particularly prevalent in these samples. Yeasts were rarely isolated from the air samples and only two species, namely, *C. pararugosa* and *Cystofilobasidium ferigula*, were identified. Species diversity in the raw material samples was also narrow, including *Saccharomyces* sensu stricto species, *W. anomalus* and *Z. rouxii*. Altogether, nine species were identified in the soft drink samples, *W. anomalus* and *Z. rouxii* being the most abundant species. Most of the strains of the six species found in the alcoholic products were identified as *C. pararugosa*, *S*. *cerevisiae*, or *W. anomalus*. *Candida magnoliae*, *Candida sojae*, *D. bruxellensis*, *Saccharomyces bayanus*, and *Naganishia liquefaciens* and *Z. bailii* were mainly linked with the soft drink products and *C. pararugosa*, *Pichia manschurica*, *Meyerozyma caribbica*, and *S. cerevisiae* with the alcoholic beverages.

Fifty‐nine independent filamentous fungi isolates were obtained from the QC samples (Figure 4b, Table S1). In total, 32 isolates were identified from air samples, three from filling line surface samples, 10 from soft drink products, 13 from beer products, and one from other alcoholic products. All of the 27 filamentous fungal species isolated from QC samples belonged to *Ascomycota* phylum. *Penicillium* (61% of the isolates) was the most common genus in the samples (Figure 4). Other genera isolated included *Phoma* (7.1%), *Talaromyces* (5.4%), *Aspergillus* (4%), *Fusarium* (4%), *Paecilomyces* (4%), *Exophiala* (4%), and *Peyronellaea* (4%). Single isolates were also identified from the genera *Didymella*, *Paecilomyces*, *Mucor*, *Cladosporium*, *Ciliciopodium*, and *Pseudogymnoascus*. The greatest number and diversity of species was detected in the air samples. Especially different *Penicillium* species were common. From soft drink samples, only *Penicillium* species were identified. Diversity of filamentous fungi in beer samples was higher and in addition to *Penicillium* species, species belonging to genera *Fusarium*, *Phoma*, *Paecilomycetes*, *Didymella*, and *Mucor* were detected. Filamentous fungi were not detected in raw material samples.

### 3.3 Spoilage ability of fermentative yeast isolates

Spoilage ability of 28 isolates of fermentative yeast species was studied in different types of alcoholic and nonalcoholic beverage products using a challenge test (Table 2). Representative strains of each species identified from the various product samples and those raw material or process isolates not identified in the product samples were included in the study. Nonfermentative species and filamentous fungi were excluded due to their expected low spoilage potential.

**TABLE 2:** Spoilage ability of selected fermentative yeast isolates in commercial beverage products as evaluated by using a challenge test

Isolate	Species	Isolation source	Turbidity development					
			Simple soft drink (sorbate, pH 2.9)	Soft drink with juice (pH 2.7)	Enhanced water (benzoate, pH 4.3)	Beer (4.5% alc., pH 4.2)	Cider (4.5% alc., sulfite, pH 3.0)	Nonbeer beverage (5.5% alc. pH 2.8)
T‐41.1	*Candida magnoliae*	Soft drink	−^a^	++	+	++	−	−
P‐57.1	*Candida parapsilosis*	Carbon dioxide	−	w	+	+	−	−
P‐3.2	*Candida picinguabensis*	Alcoholic drink	−	−	w	−	−	−
P‐2.7	*Candida* sp.	Alcoholic drink	−	−	+	+	−	−
T‐39	*Candida sojae*	Soft drink	−	++	+	++	−	−
P‐71	*Debaryomyces hansenii*	Soft drink	−	−	w	−	−	−
T‐41.5	*Dekkera bruxellensis*	Soft drink	++	+^b^	+	++	−	−
T‐47	*Dekkera bruxellensis*	Soft drink	++^c^	++	+	+	−	−
P‐3.3	*Kazachstania exigua*	Alcoholic drink	w^d^	++^e^	+	+	−	−
T‐31	*Meyerozyma caribbica*	Beer	−	++	+	+	−	−
R‐7	*Kregrevanrija delftensis*	Cider	−	+	w	w	−	−
P‐1.2	*Pichia kudriavzevii*	Alcoholic drink	w	+	++	+	−	−
T‐51	*Pichia manshurica*	Wine ingredient	−	+	+	+	++	−
P‐70	*Clavispora lusitaniae*	Carbon dioxide	−	++	+	+	−	−
T‐11	*Saccharomyces cerevisiae*	Beer	w	−	+	++	−	−
T‐30	*Saccharomyces cerevisiae*	Cider	−	+	+	+	−	−
T‐6	*Saccharomyces cerevisiae*	Alcohol ingredient	w	+	+	++	−	−
T‐12.1	*Saccharomyces cerevisiae*	Soft drink	w	+	+	+	−	−
T‐14.1	*Saccharomyces bayanaus/uvarum*	Soft drink	w	+	+	+	−	−
T‐10	*Torulaspora delbrueckii*	Beer	−	−	++	+	−	−
T‐44.2	*Wickerhamomyces anomalus*	Soft drink	−	−	++	+	−	−
T‐48	*Wickerhamomyces anomalus*	Soft drink	−	−	++	+	−	−
T‐16	*Wickerhamomyces anomalus*	Beer	−	−	++	+	−	−
T‐29	*Wickerhamomyces anomalus*	Beer	−	−	++	++	−	−
T‐9.3	*Wickerhamomyces anomalus*	Beer	−	−	+	+	−	−
P‐3.7	*Yarrowia galli*	Alcoholic drink	−	−	+	−	−	−
T‐41.2	*Zygosaccharomyces bailii*	Soft drink	+	+	+	w	++	−
T‐8.3	*Zygosaccharomyces rouxii*	Beer	w	−	w	w	−	−

*Note*: All products except enhanced water were carbonated. They were distributed in 9‐ml aliquots in 10‐ml plastic tubes and inoculated at 10^3^–10^4^ cfu ml^−1^, followed by incubation at 25°C for 6 weeks. The growth was measured weekly using turbidometry at 620 nm.

^a^ Turbidity increase less than 1.5‐fold.

^b^ Moderate growth (turbidity increase more than twofold but less than fourfold).

^c^ Intense growth (turbidity increase more than fourfold).

^d^ Weak growth (turbidity increase 1.5 to twofold).

^e^ One out of two replicates.

Turbidity increase was the main sign of yeast spoilage in clear and slightly opaque products. Visual turbidity typically appeared within 1–3 weeks after inoculation. Obvious off‐flavors, noticeable by sniffing of the products, or pH changes were not detected at the end of the 6‐week follow‐up period (data not shown). Overall, the strains of the species *D. bruxellensis*, *P. manshurica*, and *Z. bailii* showed the highest spoilage ability, growing in four out of the six studied products (Table 2). *Candida* sp. (closest to *Candida qinglingensis*), *Debaryomyces hansenii*, and *Yarrowia galli* had the lowest spoilage abilities in the studied products. *Z. rouxii* grew very weakly, if at all, in the products.

Most of the isolates were unable to cause turbidity in carbonated, chemically preserved products. Preservative‐resistant isolates growing well in at least one of the two products belonged to the species *D. bruxellensis*, *P. manschurica*, and *Z. bailii*. *D. bruxellensis*, and *Z. bailii* were able to cause heavy turbidity in the sorbate‐preserved soft drink (pH 2.9), whereas *P. manshurica* and *Z. bailii* grew well in the sulfite‐preserved cider (pH 3.0). *Kazahstania exiqua*, *Pichia kudriavzevii*, most of the *Saccharomyces* isolates and *Z. rouxii* caused slight turbidity increase in the sorbate preserved drink. The preservative‐free soft drink (pH 2.7), beer (4.5 vol‐% alcohol, pH 4.2) and the still water product with added sugar and benzoate (pH 4.3) were good growth media for most of the isolates. The alcoholic nonbeer beverage (5.5 vol‐% alcohol, pH 2.8) free of preservatives did not support yeast growth.

## 4 DISCUSSION

The making of beer and nonbeer beverages in a brewery environment is not a fully aseptic process, and despite regular cleaning, microorganisms tend to accumulate on process equipment surfaces and in the surrounding environment from raw materials and other sources (Bokulich et al., 2015; Storgårds et al., 2006; Stratford & James, 2003). Filling line machines are especially favorable niches for microbial attachment and growth due to the presence of product residues and water at ambient temperatures (Storgårds et al., 2006). In the present study, NGS was applied to characterize fungal communities building up at the key contamination sites of brewery filling lines during the production of beer or nonbeer beverages. Using qPCR as a prescreening method, fungi were detected in approximately half of the surface samples taken 1–5 days after last extensive cleaning. The qPCR may have missed low numbers of fungi on some of the surfaces, as it is not as sensitive as cultivation method. On the other hand, cultivation did not detect fungi in all qPCR‐positive samples, indicating the presence of dead or uncultivable cells. Fungi have previously been detected on brewery filling lines at variable abundancies. Using cultivation, Storgårds et al. (2006) showed that yeasts and filamentous fungi were among the first organisms to colonize surfaces after cleaning. Fungal counts on the surfaces were relatively low (~1000 cfu/cm^2^) compared with bacterial counts throughout the 8‐week follow up period. Timke et al. (2005a) detected yeast‐derived fatty acids in nearly all mature biofilm samples taken from beer bottling plants of two breweries, but in another study, FISH‐signals for eukaryotic microorganisms at two bottle conveyers were extremely low (Timke et al., 2005b).

Much of the available knowledge of fungal populations on brewery filling lines derives from culture‐dependent analyses. NGS gave a comprehensive overall picture of fungal diversity on the filling line surfaces. The fungal diversity at various sites was low (number of ASVs from 4 to 48) compared with bacterial diversity revealed in beer filling lines with NGS (number of OTUs from 71 to 376) (Priha et al., 2016) or other methods (Maifreni, Frigo, Bartolomeoli, Buiatti, Picon, et al., 2015; Timke et al., 2005b). The short and relatively conserved ITS marker gene region provides at best species group or genus level assignment and underestimates true species richness. In line with previous studies, fungal populations on the filling line surfaces were mainly composed of *Ascomycetes* (Bokulich et al., 2015; Hutzler et al., 2012; Kühle & Jespersen, 1998; Pham et al., 2011; Timke et al., 2007). NGS revealed a higher number of yeast genera than earlier reported with culture‐based methods. In part this is due to changes in nomenclature and in part could reflect the wide range of beverages produced in the studied breweries and detection of dead and uncultivable organisms using NGS. However, only limited number of genera, including *Candida*, *Saccharomyces*, *Torulaspora*, *Wickerhamomyces*, and *Zygosaccharomyces*, were detected at above 1% abundancies. Apart from *Zygosaccharomyces*, these yeast genera have been frequently identified on brewery filling line surfaces with other methods (Storgårds et al., 2006; Timke et al., 2007). *Zygosaccharomyces* yeasts are mainly associated with soft drink and wine production (Hutzler et al., 2012; Stratford & James, 2003).

NGS showed that filamentous fungi and black yeasts with *Ascomycota* affiliation dominated the fungal communities in some of the filling line sites, indicating their possible role in the microbial communities. Filamentous fungi have been shown to contribute to ecology of drinking water distribution system biofilms, for example, by providing support to the colonization of bacteria (Douterelo et al., 2018) and many ascomycetous genera and species are capable of biofilm formation (Siqueira & Lima, 2013). *Didymella*, the most often detected genus with NGS, is a plant pathogen originating from soil and infecting various plant including barley grains. It has not been linked with food or beverage spoilage (Chen et al., 2017). The black yeast *Exophiala* detected on the filling line surfaces is a common environmental fungi often associated with decaying wood, plants, and soil (Matos et al., 2002), but also detected in dirty bottles and the brewing process. *Exophiala* spp. may produce exopolysaccharides, which could promote the adherence and survival of the cells on the surfaces (Matos et al., 2002). No beverage spoilage incidents have been linked with this genus (Pitt & Hocking, 2009). Genus *Flavoplaca* that was the dominant genus detected in sample P11_B is a lichen frequently detected in Finland and can also grow in buildings and concrete (Stenroos et al., 2016) but no association with food or beverage spoilage have been detected.

The most common fungi within *Basidiomycota* phylum detected in surface samples from filling lines were affiliated to yeast genera *Hannaella* and *Malassezia*. *Malassezia* is a dominant component of the mycobiota on human skin (Amend, 2014). Recently, molecular methods have revealed that these difficult to cultivate species can be found in a diversity of habitats (Amend, 2014). Strains of the genus *Hannaella* have been isolated on the external surfaces of plants, which is a common habitat for many basidiomycetous yeasts (Kaewwichian et al., 2015).

Microbial accumulation on brewery filling lines can be affected by a multitude of factors including the design of the filling machine and the specific location within the equipment, the cleaning regimes, the products being filled as well as microbial sources from raw materials and environment (Bokulich et al., 2015; Priha et al., 2016). Although the present study was not designed to explore factors affecting the fungal community composition on filling lines, some associations between the sampling location and fungal community structure were detected. The filling line surfaces in two breweries occupied fungal communities characteristic to each brewery according to PCoA analysis. Despite relatively low percentage of the variance explained by the first two PCs, biological patterns may still revealed (Goodrich et al., 2014; Kuczynski et al., 2010). As the other two breweries were represented by one to two samples, clustering, if any, cannot be seen, and further studies are needed to understand the factors shaping fungal communities in filling lines. Bokulich et al. (2015) used molecular methods to study dispersal of fungi and bacteria throughout a North American brewery producing conventional and coolship beer. Microbial profiles revealed that many samples clustered by processing room and substrate type. Priha et al., 2016 used NGS to explore bacterial diversity in two brewery filling lines by mounting stainless steel coupons on the filling lines. Each of the filling lines showed characteristic bacterial communities, although some spatial and temporal fluctuations in the community structure were noted. Based on culture‐dependent and ‐independent analyses of bacterial diversity in mature beer bottling line biofilms, Timke et al. (2005a, 2005b) concluded that there is no typical biofilm community, not even for distinct regions of the bottling plant. However, the major wild yeast species isolated from brewery bottling line biofilms did not show differences (Timke et al., 2007).

The diversity of yeast species identified in the QC samples with the culture‐dependent method reflected the range of beer and nonbeer beverages produced in the studied breweries. It needs to be noted that each brewery used their own specific cultivation methods for isolation of fungi, which may have affected the observed species diversity. In breweries, *S. cerevisiae*, *Z. rouxii*, and *W. anomalus* were the most frequently isolated species. *S. cerevisiae* and *W. anomalus* have been reported as major contaminants in pitching yeast (Kühle & Jespersen, 1998), beer fermentation and conditioning vessels (Pham et al., 2011), and brewery filling lines (Storgårds et al., 2006; Timke et al., 2007). We detected *W. anomalus* and *Z. rouxii* from raw materials as well as final products, suggesting raw materials as an initial contamination source. *W. anomalus* is a ubiquitous species known to contaminate also beverage ingredients, whereas the association of *Z. rouxii* with spoilage of high sugar raw materials is well established (Laitila et al., 2010; Martorell et al., 2007; Stratford & James, 2003). *Saccharomyces* yeasts, on the other hand, occur widely in a brewery environment (Bokulich et al., 2015). The *Saccharomyces* isolates recovered from the QC samples grew well in beer, but showed at best limited growth in carbonated, chemically preserved beverages. *Saccharomyces* yeasts are well known beer spoilers (Hutzler et al., 2012; Kühle & Jespersen, 1998; Timke et al., 2007), whereas their ability to spoil chemically preserved beverages varies (Stratford & James, 2003). Our results are also in line with earlier findings that weakly fermenting *W. anomalus* species rarely spoils carbonated beverages or beer unless oxygen is available, but may grow in a variety of still beverages (Hutzler et al., 2012; Kühle & Jespersen, 1998; Pham et al., 2011; Timke et al., 2007). The *Z. rouxii* isolates characterized in the present study can also be considered harmless contaminants in the end products owing to their poor growth in acid and chemically preserved soft drinks and their inability to use the main carbohydrates of beer for growth (Krogerus et al., 2021). However, some strains of *Z. rouxii* can be resistant to food preservatives especially in high sugar media (Martorell et al., 2007;Stratford, 2006; Stratford & James, 2003).

The QC samples from filling line surfaces contained a greater diversity of yeast species compared with the raw material and product samples and extremophiles were not detected. This reflects the unselective nature of the filling line environment as the preservative factors are diluted in the product residues and oxygen is available for the growth of aerobic species (Maifreni et al., 2015; Timke et al., 2005a). Especially different aerobic and weakly fermenting *Candida* and *Pichia* species were common in line with previous studies (Storgårds et al., 2006; Timke et al., 2004). It is interesting to note *Pichia* species were not detected among the most abundant yeasts (>1%) on the filling line surfaces with NGS. The culture‐dependent identification and NGS analysis used different sample sets, which probably explains this result. Moreover, *Pichia* species as fast growing yeasts may have overtaken other slower growing species during enrichment of the QC samples (Hutzler et al., 2012) Contrary to earlier findings, no dominant yeast species was detected on the process surface samples. We could neither detect basidiomycetous *Rhodotorula mucilaginosa* yeast that has been considered among pioneer biofilm forming species in breweries (Riedl et al., 2019). Many of the yeast species detected on the filling line surface samples include strains that are capable of forming biofilm (Storgårds et al., 2006; Timke et al., 2007). *W. anomalus* has been considered a pioneer organism in brewery biofilms owing to its´ frequent association with biofilms and ability to form biofilm. Similarly, many *Candida* species found in the production of beer, fruit juice and fermented foods have shown propensity for biofilm formation (Storgårds et al., 2006; Timke et al., 2007; Zara et al., 2020). *Candida sojae* isolated in the present study was also shown to form biofilm, whereas *Trigonopsis cantarellii* was not (Krogerus et al., 2021). *S. cerevisiae* isolates from beer bottling lines lacked biofilm forming ability and were thought to colonize more mature biofilms (Timke et al., 2007). *Zygosaccharomyces* spp. have been found in the surface flocs of aging wines and in fruit juice processing plant biofilms and may also form biofilm on abiotic surfaces (Tristezza et al., 2010; Zara et al., 2020).

This study reinforces the previous findings that many yeast contaminants associated with beer and nonbeer beverage production are opportunistic spoilers that may grow in final products only in case of some process failure or if the contamination level overrides the efficacy of the preservative system (Stratford, 2006; Stratford & James, 2003). This was shown by the ability of many of the yeast isolates to grow in the preservative‐free soft drink. Moreover, nearly all species isolated from the QC samples grew in beer under aerobic conditions, but the growth of weakly fermenting *Candida*, *Pichia*, and *Wickerhamomyces* spp. was prevented in oxygen limited conditions (data not shown). Of the species isolated in the present study, *C. parapsilosis*, *C. sojae*, *Clavispora lusitaniae*, *P. membranifaciens*, *P. kudrivazevii*, *K. exiqua*, *T. delbrueckii*, and *W. anomalus* have been classified as commonly encountered second division, group 2 spoilage/hygiene yeast species in soft drink factories (Stratford, 2006). The results of the present study also imply that some of the new types of beverage, such as the moderately acidic, still water product containing sugar and benzoate, support the growth of opportunistic spoilers. In particular, the ubiquitous *W. anomalus* species could be a threat in this kind of beverages. Furthermore, the addition of new extract sources into beer when producing beer mix beverages could render the products susceptible to spoilage by various *Zygosaccharomyces* species.

Our results support the previous findings that the preservative resistant yeasts are relatively rare in beverage production, but if access is gained, they can cause spoilage of a variety of alcoholic and nonalcoholic drinks (Stratford, 2006). *Z. bailii* and *D. bruxellensis* were the only species growing abundantly in the sorbate preserved beverage and sulfite was tolerated only by *Pichia manschurica* and *D. bruxellensis* isolates. *D. bruxellensis* and *Z. bailii* are well documented preservative resistant yeast spoiling various alcoholic and nonalcoholic beverages (Dimopoulou et al., 2019; Hutzler et al., 2012; Martorell et al., 2007; Smith & Divol, 2016). Although *P. manshurica* has been mostly linked with spontaneous wine fermentations and wine spoilage (Perpetuini et al., 2021), it has also been found in breweries (Turvey et al., 2016). Wine spoilage isolates have shown variable resistance to sulfites and some isolates readily formed biofilm (Perpetuini et al., 2021; Tristezza et al., 2010).

Common spoiling filamentous fungi originating usually from outdoor air are species from genus *Aspergillus* and *Penicillium* that were also the most common filamentous fungi detected in the QC samples together with species from genera *Talaromyces* and *Phoma*. *Talaromyces* spp. are heat‐resistant filamentous fungi usually of soil origin and are frequently detected in pasteurized fruit‐based products including flavored mineral waters (Hocking & Pitt, 2001; Pitt & Hocking, 2009). Species from genus *Phoma* are common outdoor and soil fungi that have been previously detected also in soft drink manufacturing facilities (Aoyama & Miyamoto, 2016).

Filamentous fungi usually require oxygen to grow. One exception is *Paecilomyctes variotii* which was detected in QC samples and can grow in microanaerobic conditions (Wareing, 2016). However, it is rarely able to grow in carbonated products. *Paecilomycetes* genus is an anamorph of heat‐resistant ascomycete genus *Byssochlamys* (Samson et al., 2009). Some filamentous fungi produce mycotoxins that cause a food safety concern for humans. *P. variotii* is able to produce mycotoxin viriditoxin and *Fusarium oxysporum* that was also detected in QC samples produces oxysporone that is commonly detected in treated orange juice (Wareing, 2016). In addition to mycotoxin production, raw material contamination with *Fusarium* species can also lead to production of gushing inducers and gushing of beer have been detected in beers produced from malts contaminated with *Fusarium* species (Sarlin et al., 2005; Sarlin et al., 2007).

## 5 CONCLUSIONS

This study explored fungal diversity in modern breweries producing beer and nonbeer beverages. NGS analysis was found to be a good tool for obtaining a comprehensive view of fungal communities on filling line surfaces. It revealed higher number of fungal genera in association with brewery filling lines than earlier reported when applying other methods. The fungal species identified in QC samples reflected the range of beer and nonbeer beverages produced in the studied breweries. Majority of the isolated yeast contaminants did not appear to pose a spoilage threat in carbonated, chemically preserved beverages or in beer but could play a role in the establishment of biofilm on equipment surfaces or in the spoilage of new types of beverage products. Preservative resistant species were rare. This study underlies the importance of maintaining good process hygiene especially when producing beverages with reduced hurdles. The findings of the study may be applied to evaluate harmfulness of fungal contaminants detected in breweries.

## CONFLICT OF INTEREST

PBL Brewing Laboratory funded this research. The authors declare that the research was conducted in the absence of any commercial or financial relationships that could be construed as a potential conflict of interest.

## Supporting information


**Table S1.** Yeast and filamentous fungi identified in quality control samples using Sanger sequencing with fungal D1/D2 and ITS region primers.

**Figure S1.** Fungal phyla present in brewery bottling and canning line surfaces as determined by NGS. Phyla detected under 1% relative abundance are grouped together.

**Figure S2.** Fungal alpha diversity in brewery bottling and canning line surfaces. Number of observed taxonomic units (ASVs), estimated number of ASVs (Chao1) and Shannon diversity index are presented.

## References

[yea3687-bib-0001] Amend, A. (2014). From dandruff to deep‐sea vents: Malassezia‐like fungi are ecologically hyper‐diverse. PLoS Pathogens, 10(8), e1004277. 10.1371/journal.ppat.1004277 25144294 PMC4140847

[yea3687-bib-0002] Aoyama, F. , & Miyamoto, T. (2016). Development of a DNA array for the simple identification of major filamentous fungi in the beverage manufacturing environment. Biocontrol Science, 21(3), 161–172. 10.4265/BIO.21.161 27667521

[yea3687-bib-0003] Begrow, W. (2017). Fighting quality threats: Notable microbiological contaminations of craft beer in the United States. Brewing and Beverage Industry International, 5, 3–10.

[yea3687-bib-0004] Bokulich, N. A. , Bergsveinson, J. , Ziola, B. , & Mills, D. A. (2015). Mapping microbial ecosystems and spoilage‐gene flow in breweries highlights patterns of contamination and resistance. eLife, 2015(4), e04634. 10.7554/ELIFE.04634 PMC4352708 25756611

[yea3687-bib-0005] Callahan, B. J. , McMurdie, P. J. , Rosen, M. J. , Han, A. W. , Johnson, A. J. A. , & Holmes, S. P. (2016). DADA2: High‐resolution sample inference from Illumina amplicon data. Nature Methods, 13(7), 581–583. 10.1038/nmeth.3869 27214047 PMC4927377

[yea3687-bib-0006] Chao, A. (1984). Nonparametric estimation of the number of classes in a population. Scandinavian Journal of Statistics, 11(4), 265–270. https://www.jstor.org/stable/4615964

[yea3687-bib-0007] Chen, Q. , Hou, L. W. , Duan, W. J. , Crous, P. W. , & Cai, L. (2017). Didymellaceae revisited. Studies in Mycology, 87, 105–159. 10.1016/J.SIMYCO.2017.06.002 28706324 PMC5498420

[yea3687-bib-0008] Davenport, R. R. (1996). Forensic microbiology for the soft drinks business. In Soft drinks management international (pp. 34–35).

[yea3687-bib-0009] Dimopoulou, M. , Hatzikamari, M. , Masneuf‐Pomarede, I. , & Albertin, W. (2019). Sulfur dioxide response of *Brettanomyces bruxellensis* strains isolated from Greek wine. Food Microbiology, 78, 155–163. 10.1016/j.fm.2018.10.013 30497597

[yea3687-bib-0010] Douterelo, I. , Calero‐Preciado, C. , Soria‐Carrasco, V. , & Boxall, J. B. (2018). Whole metagenome sequencing of chlorinated drinking water distribution systems. Environmental Science: Water Research & Technology, 4(12), 2080–2091. 10.1039/C8EW00395E

[yea3687-bib-0011] Filtenborg, O. , Frisvad, J. C. , & Samson, R. A. (2004). Specific association of fungi to foods and influence of physical environmental factors. Introduction to Food‐ and Airborne Fungi, Ed.7, 306–320.

[yea3687-bib-0012] Gardes, M. , & Bruns, T. D. (1993). ITS primers with enhanced specificity for basidiomycetes—Application to the identification of mycorrhizae and rusts. Molecular Ecology, 2(2), 113–118. 10.1111/J.1365-294X.1993.TB00005.X 8180733

[yea3687-bib-0013] Goodrich, J. K. , Di Rienzi, S. C. , Poole, A. C. , Koren, O. , Walters, W. A. , Caporaso, J. G. , Knight, R. , & Ley, R. E. (2014). Conducting a microbiome study. Cell, 158(2), 250–262. 10.1016/J.CELL.2014.06.037 25036628 PMC5074386

[yea3687-bib-0014] Haugland, R. , & Vesper, S. (2002). Method of identifying and quantifying specific fungi and bacteria.

[yea3687-bib-0015] Hocking, A. , & Pitt, J. I. (2001). Moulds. In C. Moir , C. Anderw‐Kabilafkas , G. Arnold , B. Cox , H. A. D. Hocking , & I. Jensen (Eds.), Spoilage of processed foods: causes and diagnosis (pp. 361–281). AIFST Inc (NSW Branch) Food Microbiology Group.

[yea3687-bib-0016] Hutzler, M. , Riedl, R. , Koob, J. , & Jacob, F. (2012). Fermentation and spoilage yeasts and their relevance for the beverage industry—A review. BrewingScience, 65(3–4), 33–52. https://www.researchgate.net/publication/287235582

[yea3687-bib-0017] Hutzler, M. , Wellhoener, U. , Tenge, C. , & Geiger, E. (2008). Beer mixed beverages: dan gerous spoilage yeasts, susceptible beverages? Brauwelt International, 26(4), 206–211.

[yea3687-bib-0018] Juvonen, R. , Virkajärvi, V. , Priha, O. , & Laitila, A. (2011). Microbiological spoilage and safety risks in non‐beer beverages. VTT Research notes.

[yea3687-bib-0019] Kaewwichian, R. , Jindamorakot, S. , Am‐In, S. , Sipiczki, M. , & Limtong, S. (2015). Hannaella siamensis sp. nov. and *Hannaella phetchabunensis* sp. nov., two new anamorphic basidiomycetous yeast species isolated from plants. International Journal of Systematic and Evolutionary Microbiology, 65(Pt_4), 1297–1303. 10.1099/IJS.0.000101 25644481

[yea3687-bib-0020] Kõljalg, U. , Nilsson, R. H. , Abarenkov, K. , Tedersoo, L. , Taylor, A. F. S. , Bahram, M. , Bates, S. T. , Bruns, T. D. , Bengtsson‐Palme, J. , Callaghan, T. M. , Douglas, B. , Drenkhan, T. , Eberhardt, U. , Dueñas, M. , Grebenc, T. , Griffith, G. W. , Hartmann, M. , Kirk, P. M. , Kohout, P. , … Larsson, K. H. (2013). Towards a unified paradigm for sequence‐based identification of fungi. In Molecular ecology (Vol. 22, Issue 21) (pp. 5271–5277). John Wiley & Sons, Ltd.. 10.1111/mec.12481 24112409

[yea3687-bib-0021] Krogerus, K. , Eerikäinen, R. , Aisala, H. , & Gibson, B. (2021). Repurposing brewery contaminant yeast as production strains for low‐alcohol beer fermentation. Yeast. 10.1002/yea.3674 34664308

[yea3687-bib-0022] Kuczynski, J. , Liu, Z. , Lozupone, C. , McDonald, D. , Fierer, N. , & Knight, R. (2010). Microbial community resemblance methods differ in their ability to detect biologically relevant patterns. Nature Methods, 7(10), 813–819. 10.1038/nmeth.1499 20818378 PMC2948603

[yea3687-bib-0023] Kühle, A. , & Jespersen, L. (1998). Detection and identification of wild yeasts in lager breweries. International Journal of Food Microbiology, 43, 205–213. 10.1016/S0168-1605(98)00113-5 9801196

[yea3687-bib-0024] Kurtzman, C. P. , & Robnett, C. J. (1997). Identification of clinically important ascomycetous yeasts based on nucleotide divergence in the 5′ end of the large‐subunit (26S) ribosomal DNA gene. Journal of Clinical Microbiology, 35(5), 1216–1223. 10.1128/JCM.35.5.1216-1223.1997 9114410 PMC232732

[yea3687-bib-0025] Laitila, A. , Sarlin, T. , Raulio, M. , Wilhelmson, A. , Kotaviita, E. , Huttunen, T. , & Juvonen, R. (2010). Yeasts in malting, with special emphasis on *Wickerhamomyces anomalus* (synonym *Pichia anomala*). Antonie Van Leeuwenhoek, 99(1), 75–84. 10.1007/S10482-010-9511-8 20872177

[yea3687-bib-0026] Maifreni, M. , Frigo, F. , Bartolomeoli, I. , Buiatti, S. , Picon, S. , & Marino, M. (2015). Bacterial biofilm as a possible source of contamination in the microbrewery environment. Food Control, 50, 809–814. 10.1016/J.FOODCONT.2014.10.032

[yea3687-bib-0027] Martin, M. (2011). Cutadapt removes adapter sequences from high‐throughput sequencing reads. EMBnet. Journal, 17(1), 10. 10.14806/ej.17.1.200

[yea3687-bib-0028] Martorell, P. , Stratford, M. , Steels, H. , Fernández‐Espinar, M. T. , & Querol, A. (2007). Physiological characterization of spoilage strains of *Zygosaccharomyces bailii* and *Zygosaccharomyces rouxii* isolated from high sugar environments. International Journal of Food Microbiology, 114(2), 234–242. 10.1016/j.ijfoodmicro.2006.09.014 17239464

[yea3687-bib-0029] Matos, T. , De Hoog, G. S. , De Boer, A. G. , De Crom, I. , & Haase, G. (2002). High prevalence of the neurotrope *Exophiala dermatitidis* and related oligotrophic black yeasts in sauna facilities. Mycoses, 45(9–10), 373–377. 10.1046/j.1439-0507.2002.00779.x 12421284

[yea3687-bib-0030] McMurdie, P. J. , & Holmes, S. (2013). phyloseq: An R package for reproducible interactive analysis and graphics of microbiome census data. PLoS ONE, 8(4), e61217. 10.1371/journal.pone.0061217 23630581 PMC3632530

[yea3687-bib-0031] Morgulis, A. , Coulouris, G. , Raytselis, Y. , Madden, T. , Agarwala, R. , & Schäffer, A. (2008). Database indexing for production MegaBLAST searches. Bioinformatics (Oxford, England), 24(16), 1757–1764. 10.1093/BIOINFORMATICS/BTN322 PMC2696921 18567917

[yea3687-bib-0032] Perpetuini, G. , Rossetti, A. P. , Battistelli, N. , Arfelli, G. , & Tofalo, R. (2021). Adhesion properties, biofilm forming potential, and susceptibility to disinfectants of contaminant wine yeasts. Microorganisms, 9, 654. 10.3390/MICROORGANISMS9030654 33809953 PMC8004283

[yea3687-bib-0033] Pham, T. , Wimalasena, T. , Box, W. G. , Koivuranta, K. , Storgårds, E. , Smart, K. A. , & Gibson, B. R. (2011). Evaluation of ITS PCR and RFLP for differentiation and identification of brewing yeast and brewery ‘wild’ yeast contaminants. Journal of the Institute of Brewing, 117(4), 556–568. 10.1002/J.2050-0416.2011.TB00504.X 32834175 PMC7197508

[yea3687-bib-0034] Pitt, J. I. , & Hocking, A. D. (2009). Fungi and food spoilage. Springer. 10.1007/978-0-387-92207-2

[yea3687-bib-0035] Priha, O. , Raulio, M. , Maukonen, J. , Vehviläinen, A. K. , & Storgårds, E. (2016). Bacterial populations on brewery filling hall surfaces as revealed by next‐generation sequencing. Biofouling, 32(5), 571–581. 10.1080/08927014.2016.1154946 27064426

[yea3687-bib-0036] Riedl, R. , Fütterer, J. , Goderbauer, P. , Michel, M. , Jacob, F. , & Hutzler, M. (2019). Combined yeast biofilm screening–characterization and validation of yeast related biofilms in a brewing environment with combined cultivation and specific real‐time PCR screening of selected indicator species. Journal of the American Society of Brewing Chemists, 77(2), 99–112. 10.1080/03610470.2019.1579036

[yea3687-bib-0061] RStudio Team . (2021). RStudio: Integrated development for R. RStudio, PBC, Boston, MA. http://www.rstudio.com/

[yea3687-bib-0037] Samson, R. A. , Houbraken, J. , Varga, J. , & Frisvad, J. C. (2009). Polyphasic taxonomy of the heat resistant ascomycete genus *Byssochlamys* and its *Paecilomyces* anamorphs. Persoonia, 22, 14–27. 10.3767/003158509X418925 20198134 PMC2789542

[yea3687-bib-0038] Sarlin, T. , Nakari‐Setälä, T. , Linder, M. , Penttilä, M. , & Haikara, A. (2005). Fungal hydrophobins as predictors of the gushing activity of malt. Journal of the Institute of Brewing, 111(2), 105–111. 10.1002/J.2050-0416.2005.TB00655.X

[yea3687-bib-0039] Sarlin, T. , Vilpola, A. , Kotaviita, E. , Olkku, J. , & Haikara, A. (2007). Fungal hydrophobins in the barley‐to‐beer chain. Journal of the Institute of Brewing, 113(2), 147–153. 10.1002/J.2050-0416.2007.TB00271.X

[yea3687-bib-0040] Shannon, C. E. (1948). A mathematical theory of communication. Bell System Technical Journal, 27(379–423), 623–656. 10.1002/j.1538-7305.1948.tb01338.x

[yea3687-bib-0041] Siqueira, V. M. , & Lima, N. (2013). Biofilm formation by filamentous fungi recovered from a water system. Journal of Mycology, 2013, 1–9. 10.1155/2013/152941

[yea3687-bib-0042] Smith, B. D. , & Divol, B. (2016). *Brettanomyces bruxellensis*, a survivalist prepared for the wine apocalypse and other beverages. In Food microbiology (Vol. 59) (pp. 161–175). Academic Press. 10.1016/j.fm.2016.06.008 27375257

[yea3687-bib-0043] Stenroos, S. , Velmala, S. , Pykälä, J. , & Ahti, T. (2016). Lichens of Finland. Botanical Museum, Finnish Museum of Natural History.

[yea3687-bib-0044] Storgårds, E. , & Priha, O. (2009). Biofilms and brewing. In Biofilms in the food and beverage industries (pp. 432–454). Elsevier Inc.. 10.1533/9781845697167.4.432

[yea3687-bib-0045] Storgårds, E. , Tapani, K. , Hartwall, P. , Saleva, R. , & Suihko, M. L. (2006). Microbial attachment and biofilm formation in brewery bottling plants. Journal of the American Society of Brewing Chemists, 64(1), 8–15. 10.1094/ASBCJ-64-0008

[yea3687-bib-0046] Stratford, M. (2006). Food and beverage spoilage yeasts. In G. Querol & H. Fleet (Eds.), Yeasts in food and beverages. Springer‐Verlag. 10.1007/978-3-540-28398-0_11.pdf

[yea3687-bib-0047] Stratford, M. , & James, S. (2003). Non‐alcoholic beverages and yeasts. In T. Boekhout & V. Robert (Eds.), Yeasts in food (pp. 309–345). Behr's Verlag GmbH & Co. Hutzler. 10.1533/9781845698485.309

[yea3687-bib-0048] Timke, M. , Wang‐Lieu, N. Q. , Altendorf, K. , & Lipski, A. (2005a). Fatty acid analysis and spoilage potential of biofilms from two breweries. Wiley Online Library, 99(5), 1108–1122. 10.1111/j.1365-2672.2005.02714.x 16238741

[yea3687-bib-0049] Timke, M. , Wang‐Lieu, N. Q. , Altendorf, K. , & Lipski, A. (2005b). Community structure and diversity of biofilms from a beer bottling plant as revealed using 16S rRNA gene clone libraries. Applied and Environmental Microbiology, 71(10), 6446–6452. 10.1128/AEM.71.10.6446-6452.2005 16204578 PMC1266004

[yea3687-bib-0050] Timke, M. , Wang‐Lieu, N. Q. , Altendorf, K. , & Lipski, A. (2007). Identity, beer spoiling and biofilm forming potential of yeasts from beer bottling plant associated biofilms. Antonie Van Leeuwenhoek, 93(1), 151–161. 10.1007/S10482-007-9189-8 17659449

[yea3687-bib-0051] Timke, M. , Wolking, D. , Wang‐Lieu, N. Q. , Altendorf, K. , & Lipski, A. (2004). Microbial composition of biofilms in a brewery investigated by fatty acid analysis, fluorescence in situ hybridisation and isolation techniques. Applied Microbiology and Biotechnology, 66(1), 100–107. 10.1007/s00253-004-1601-y 15085296

[yea3687-bib-0052] Tribst, A. A. L. , De Souza Sant́ana, A. , & De Massaguer, P. R. (2009). Review: Microbiological quality and safety of fruit juicespast, present and future perspectives Microbiology of fruit juices. Critical Reviews in Microbiology, 35(4), 310–339. 10.3109/10408410903241428 19863382

[yea3687-bib-0053] Tristezza, M. , Lourenço, A. , Barata, A. , Brito, L. , Malfeito‐Ferreira, M. , & Loureiro, V. (2010). Susceptibility of wine spoilage yeasts and bacteria in the planktonic state and in biofilms to disinfectants. Annals of Microbiology, 60(3), 549–556. 10.1007/S13213-010-0085-5

[yea3687-bib-0054] Turvey, M. E. , Weiland, F. , Meneses, J. , Sterenberg, N. , & Hoffmann, P. (2016). Identification of beer spoilage microorganisms using the MALDI Biotyper platform. Applied Microbiology and Biotechnology, 100(6), 2761–2773. 10.1007/s00253-016-7344-8 26857464

[yea3687-bib-0055] van Esch, F. (1987). Yeasts in soft drinks and fruit juice concentrates. Warenchemicus, 17, 20–31.

[yea3687-bib-0056] Wareing, P. (2016). Microbiology of soft drinks and fruit juices. In P. R. Ashurst (Ed.), Chemistry and technology of soft drinks and fruit juices (pp. 345–366). Wiley‐Blackwell. 10.1002/9781118634943.ch11

[yea3687-bib-0057] White, T. , Bruns, T. , Lee, S. , & Taylor, P. (1990). Amplification and direct sequencing of fungal ribosomal RNA genes for phylogenetics. PCR Protocols: A Guide to Methods and Applications, 18(1), 315–322. 10.1016/B978-0-12-372180-8.50042-1

[yea3687-bib-0058] Wickham, M. H. (2015). Package “ggplot2” Type Package Title An implementation of the Grammar of Graphics.

[yea3687-bib-0059] Zara, G. , Budroni, M. , Mannazzu, I. , Fancello, F. , & Zara, S. (2020). Yeast biofilm in food realms: occurrence and control. World Journal of Microbiology and Biotechnology, 36(9), 1–10. 10.1007/S11274-020-02911-5 PMC7415760 32776210

