# Supplementary material for: Fungal diversity on brewery filling hall surfaces and quality control samples
Source: Yeast. 2022 Jan 12;39(1-2):141–55. doi: 10.1002/yea.3687 (PMC9303908; doi:10.1002/yea.3687)
Supplement: Supplementary file 1 — Table S1. Yeast and filamentous fungi identified in quality control samples using Sanger sequencing with fungal D1/D2 and ITS region primers. [file YEA-39-141-s003.docx]

**Supplementary data:**

Figure S 1. Fungal phyla present in brewery bottling and canning line surfaces as determined by NGS. Phyla detected under 1% relative abundance are grouped together.

Figure S 2. Fungal alpha diversity in brewery bottling and canning line surfaces. Number of observed taxonomic units (ASVs), estimated number of ASVs (Chao1) and Shannon diversity index are presented.

Table S 1.  Yeast and filamentous fungi identified in quality control samples using Sanger sequencing with fungal D1/D2 and ITS region primers.

| **Sample ID** | **Sample**  **category** | **Yeast species** | **Mould species** |
| --- | --- | --- | --- |
| I-1 | air |  | *Penicillium expansum* |
|  |  |  | *Penicillium commune* |
|  |  |  | *Peyronellellaea arachidicola* |
|  |  |  | *Penicillium expansum* |
|  |  |  | *Penicillium digitatum* |
| I-2 | air |  | *Penicillium expansum* |
|  |  |  | *Penicillium bussumense/glabrum* |
| I-3 | air |  | *Fusarium oxysporum* |
|  |  |  | *Penicillium brevicompactum* |
|  |  |  | *Penicillium corylophilum* |
|  |  |  | *Penicillium expansum* |
| I-4 | air |  | *Penicillium expansum* |
| I-5 | air |  | *Penicillium brevicompactum* |
|  |  |  | *Cladosporium sphaerospermum* |
|  |  |  | *Penicillium commune* |
|  |  |  | *Penicillium decumbens* |
|  |  |  | *Penicillium brevicompactum* |
|  |  |  | *Peyronellaea arachidicola* |
| I-9 | air | *Cystofilobasidium ferigula* | *Penicillium brevicompactum* |
| I-10 | air | *Candida pararugosa* | *Talaromyces sp.* |
|  |  |  | *Aspergillus pseudoglaucus* |
|  |  |  | *Talaromyces ruber* |
| I-12 | air |  | *Talaromyces radicus* |
|  |  |  | *Penicillium brevicompactum* |
|  |  |  | *Aspergillus pseudoglaucus* |
| I-15 | air |  | *Penicillium bussumense/glabrum* |
|  |  |  | *Pseudogymnoascus sp.* |
| I-16 | air |  | *Phoma* sp. |
|  |  |  | *Penicillium solitum* |
| I-37 | air |  | *Phoma* sp. |
|  |  |  | *Penicillium expansum* |
|  |  |  | *Penicillium cravenianum* |
| P-1 | filling line surface | *Saccharomyces cerevisiae* |  |
|  |  | *Pichia kudriavzevii* |  |
|  |  | *Pichia manshurica* |  |
| P-2 | filling line surface | *Pichia membranifaciens* |  |
|  |  | *Candida pararugosa* |  |
|  |  | *Wickerhamomyces anomalus* |  |
| P-3 | filling line surface | *Candida* sp*.* (closest to *qinglingensis)* |  |
|  |  | *Candida picinguabensis* |  |
|  |  | *Kazachstania exigua* |  |
|  |  | *Wickerhamomyces anomalus* |  |
|  |  | *Pichia membranifaciens* |  |
|  |  | *Yarrowia galli* |  |
| P-4 | filling line surface | - | *Penicillium crustosum* |
| P-5 | filling line surface | *Pichia kudriavzevii* |  |
|  |  | *Pichia manshurica* |  |
|  |  | *Saccharomyces cerevisiae* |  |
|  |  | *Trigonopsis variabilis* |  |
| P-40 | filling line surface | - | *Phoma* sp. |
| P-41 | filling line surface | - | *Phoma* sp. |
| P-57 | filling line surface | *Candida parapsilosis* |  |
| P-69 | filling line surface | *Trigonopsis cantarellii* |  |
| P-70 | filling line surface | *Clavispora lusitaniae* |  |
| P-71 | filling line surface | *Debaryomyces hansenii* |  |
| R-1 | raw material | *Wickerhamomyces anomalus* |  |
| R-2 | raw material | *Wickerhamomyces anomalus* |  |
| R-3 | raw material | *Wickerhamomyces anomalus* |  |
|  |  | *Zygosaccharomyces rouxii* |  |
| R-4 | raw material | *Wickerhamomyces anomalus* |  |
| R-5 | raw material | *Wickerhamomyces anomalus* |  |
| R-6 | raw material | *Saccharomyces bayanus* |  |
|  |  | *Saccharomyces cerevisiae*  *Saccharomyces eubayanus/ pastorianus* |  |
| R-7 | raw material | *Kregervanrija delftensis* |  |
| T-1 | beer | - | *Penicillium crustosum* |
| T-2 | beer | - | *Penicillium cravenianum* |
| T-4 | beer | - | *Mucor* sp. |
| T-6 | other alcoholic | *Saccharomyces cerevisiae* | *Penicillium expansum* |
| T-7 | beer | - | *Penicillium crustosum* |
| T-8 | soft drink | *Zygosaccharomyces rouxii* | *Paecilomyces variotii* |
| T-9 | soft drink | *Wickerhamomyces anomalus* | *Phoma* sp. |
|  |  | *Zygosaccharomyces rouxii* | *Fusarium oxysporum* |
| T-10 | beer | *Torulaspora delbrueckii* |  |
| T-11 | beer | *Saccharomyces cerevisiae* |  |
| T-12 | soft drink | *Saccharomyces cerevisiae* |  |
| T-13 | soft drink | - | *Penicillium commune* |
| T-14 | soft drink | *Saccharomyces bayanus* |  |
| T-15 | beer | - | *Penicillium paneum* |
| T-16 | beer | *Wickerhamomyces anomalus* |  |
| T-17 | beer | *Wickerhamomyces anomalus* |  |
| T-18 | beer | - | *Didymella pinodes* |
| T-19 | beer | *Saccharomyces cerevisiae* |  |
| T-20 | soft drink | *Wickerhamomyces anomalus* |  |
| T-26 | soft drink | *Wickerhamomyces anomalus* |  |
| T-28 | soft drink | *Naganishia liquefaciens* |  |
| T-29 | beer | *Wickerhamomyces anomalus* |  |
| T-30 | other alcoholic | *Saccharomyces cerevisiae* |  |
| T-31 | beer | *Meyerozyma caribbica* |  |
| T-32 | beer |  | *Penicillium commune* |
|  |  |  | *Penicillium crustosum* |
| T-33 | beer |  | *Penicillium paneum* |
|  |  |  | *Penicillium expansum* |
| T-34 | beer |  | *Penicillium corylophilum* |
|  |  |  | *Penicillium expansum* |
|  |  |  | *Penicillium decumbens* |
| T-35 | beer | *Candida pararugosa* |  |
| T-36 | soft drink |  | *Penicillium* sp. |
| T-38 | soft drink |  | *Exophiala dermatitidis* |
| T-39 | soft drink | *Candida sojae* |  |
| T-41 | soft drink | *Candida magnoliae* | *Penicillium cravenianum* |
|  |  | *Zygosaccharomyces bailii* | *Exophiala dermatitidis* |
|  |  | *Zygosaccharomyces rouxii* |  |
|  |  | *Dekkera bruxellensis* |  |
|  |  | *Wickerhamomyces anomalus* |  |
| T-42 | soft drink |  | *Penicillium cravenianum* |
| T-45 | soft drink |  | *Penicillium cravenianum* |
| T-49 | other alcoholic | *Candida pararugosa* |  |
| T-50 | soft drink | *Zygosaccharomyces bailii* |  |
| T-51 | other alcoholic | *Pichia manshurica* |  |
