# Supplementary figures and images for: Fungal diversity on brewery filling hall surfaces and quality control samples
Source: Yeast. 2022 Jan 12;39(1-2):141–55. doi: 10.1002/yea.3687 (PMC9303908; doi:10.1002/yea.3687)

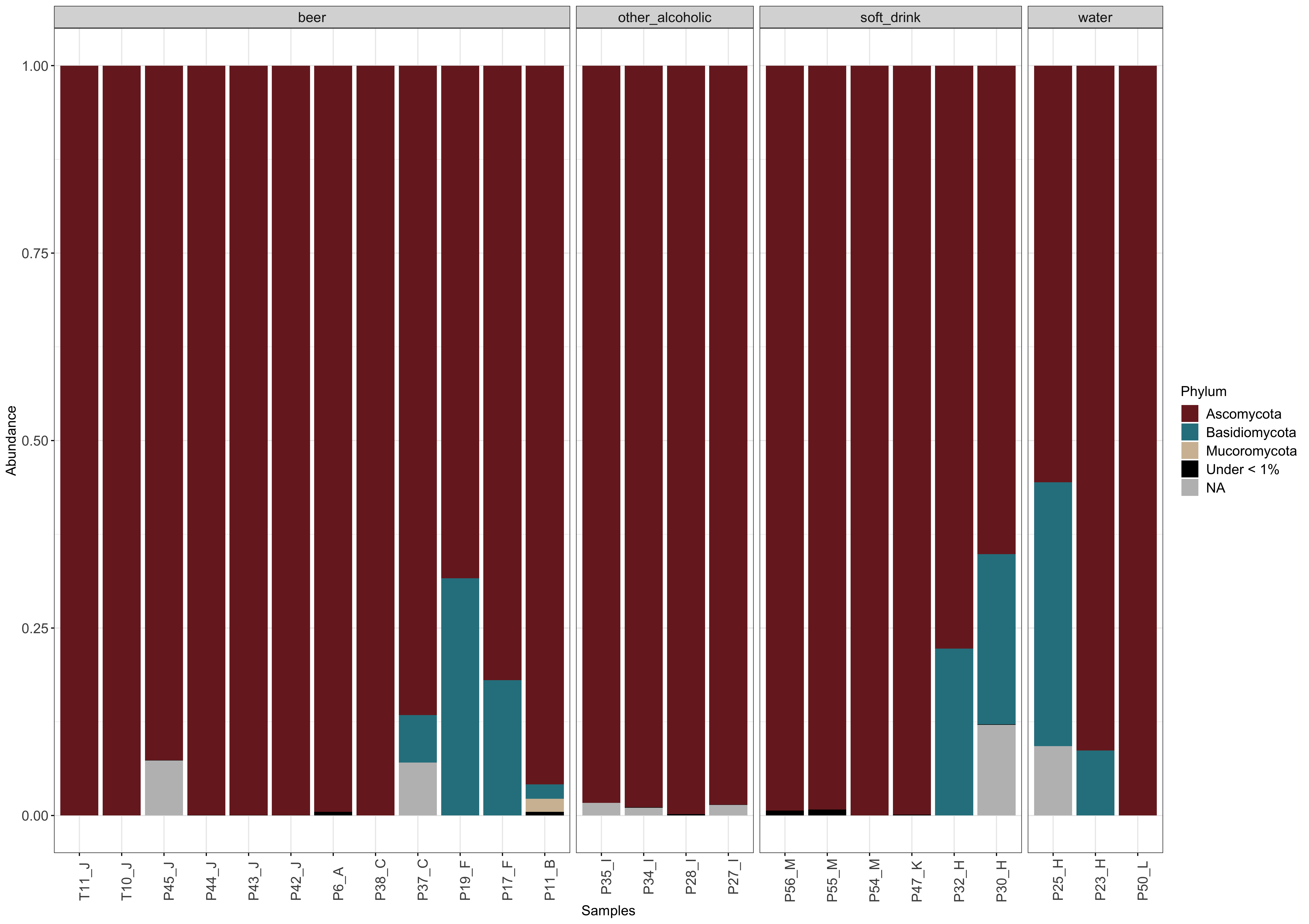

Supplement: Supplementary file 2 — Figure S1. Fungal phyla present in brewery bottling and canning line surfaces as determined by NGS. Phyla detected under 1% relative abundance are grouped together. [file YEA-39-141-s002.png]

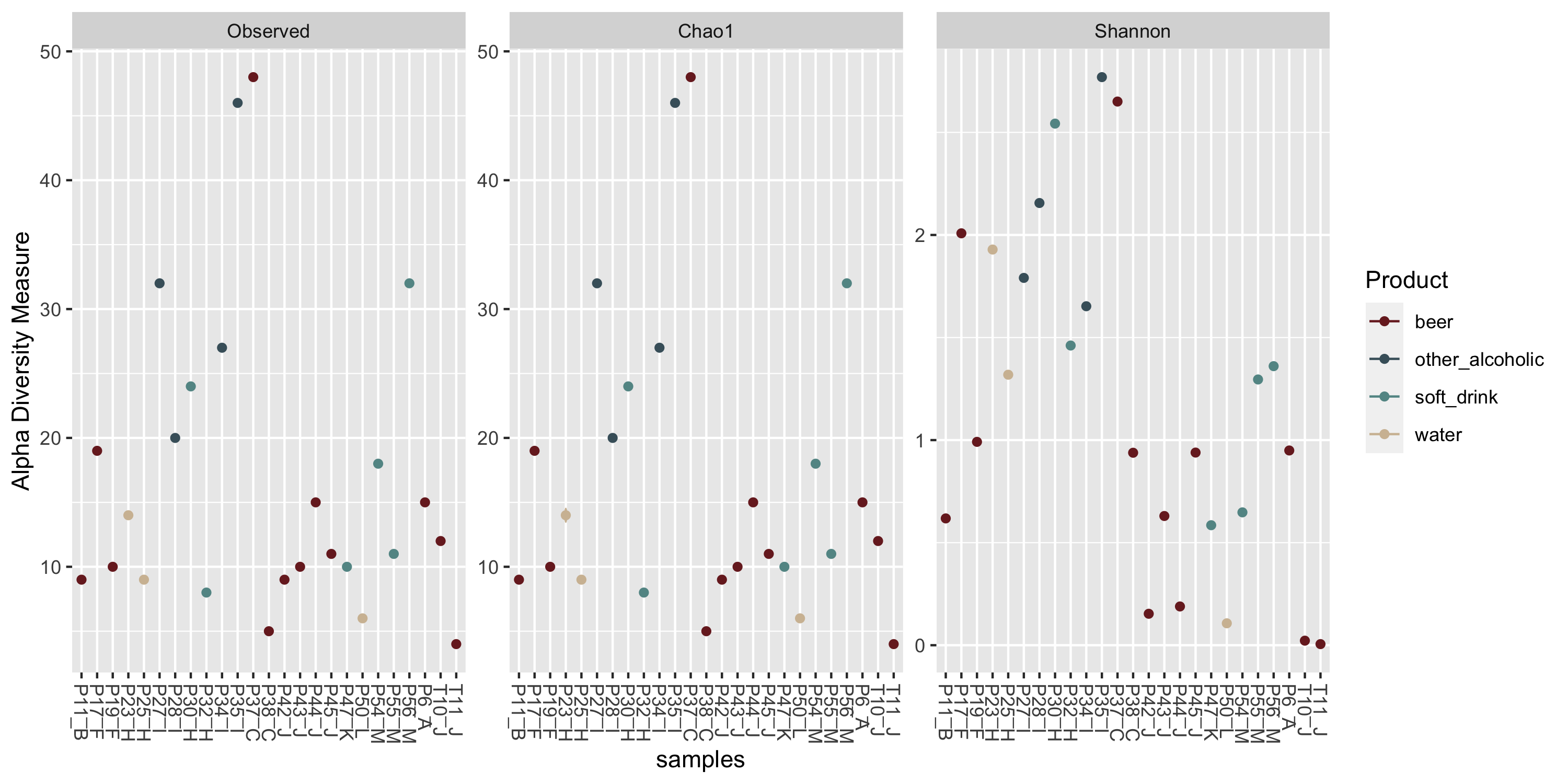

Supplement: Supplementary file 3 — Figure S2. Fungal alpha diversity in brewery bottling and canning line surfaces. Number of observed taxonomic units (ASVs), estimated number of ASVs (Chao1) and Shannon diversity index are presented. [file YEA-39-141-s001.png]
